# Supplementary material for: Integrated microRNA–mRNA Expression Profiling Identifies Novel Targets and Networks Associated with Autism
Source: J Pers Med. 2022 Jun 1;12(6):920. doi: 10.3390/jpm12060920 (PMC9225282; doi:10.3390/jpm12060920)
Supplement: Supplementary file 1 [file jpm-12-00920-s001.zip › Supplementary Table S1--S2.pdf]

Journal of
*Personalized
Medicine*

MDPI

Supplementary Materials and Methods:

Supplementary Table S1: qRT-PCR TaqMan gene expression assays for *AUTS2, FMR1, GAPDH,* and *PTEN* (ThermoFisher Scientific, Carlsbad, CA).

Entrez Gene ID	Taqman Assays	Assay ID
26053	AUTS2	Hs01688766_m1
2332	FMR1	Hs00924547_m1
2597	GAPDH	Hs02786624_g1
5728	PTEN	Hs02621230_s1

Supplementary Table S2: qRT-PCR miRNA Primer miScript Assays for *Hsa-miR-15a-5*, *Hsa-miR-92a-3p*, and *Hsa-miR-125b-5p*, *Hs_RNU6* and *Ce_miR39_1* (Qiagen, Valencia, CA).

Entrez Gene ID	SYBR Green Assays	Catalog number
406948	Hsa-miR-15a-5p	MS00003178
407048	Has-miR-92a-3p	MS00006594
406911	Hsa-miR-125b-5p	MS00006629
	Ce_miR39_1	MS00019789
26827	Hs_RNU6	MS00033740

Supplementary Table S3: RNA -Sequencing (RNA-Seq) analysis in LCL groups (ASD and Control). LC Sciences (Houston, TX, USA), Gene symbol, EntrezID, fold=fold change, pv = *p*-value and FDR= False discovery rate, *P ≤ 0.05.

Supplementary Table S4: Small RNA -Sequencing (miRNA-Seq) analysis in LCL groups (ASD and Control), LC Sciences (Houston, TX, USA). miRNA, Accession MIMATid, fold=fold change, pv = *p*-value and FDR= False discovery rate, *P ≤ 0.05.

Supplementary Table S5: Ingenuity Pathway Analysis on 1700 mRNAs (Functions & Pathways) in LCL groups (ASD and Control).

Supplementary Table S6: Ingenuity Pathway Analysis on 910DEGs-allmiRNAs-Functions -pathways in LCL groups (ASD and Control). DEGs=Differentially Expressed Genes, allmiRNAs= All microRNAs.

Supplementary Table S7: Integrated miRNA-mRNA analysis using miRWalk2.0. Ensembl, Gene, FC=Fold Change, Pathways regulated, miR= miRNA (upmiR=upregulated miRNA or downmiRs=downregulated miRNAs).
